# *Pasteurella canis* Isolation following Penetrating Eye Injury: A Case Report

**DOI:** 10.1155/2012/362369

**Published:** 2012-04-23

**Authors:** Noor-Khairul Rashid, Zarifah Zam, Siti-Suraya MdNoor, Ishak Siti-Raihan, Yaakub Azhany

**Affiliations:** ^1^Department of Ophthalmology, School of Medical Sciences, Universiti Sains Malaysia, Kubang Kerian, Kelantan 16150, Malaysia; ^2^Department of Pathology and Microbiology, School of Medical Sciences, Universiti Sains Malaysia, Kubang Kerian, Kelantan 16150, Malaysia

## Abstract

A 3-year-old boy presented with history of trauma to the left eye after he accidentally injured his eye with a broom stick made up from coconut skewers. There was history of cats as their pets but not dogs. Ocular examination revealed left superonasal conjunctival laceration and scleral perforation with prolapsed vitreous. Fundus examination showed minimal vitreous haemorrhage and flat retina. Conjunctiva swab at the wound site was sent for gram staining, culture, and sensitivity. He underwent scleral suturing, vitreous tap, and intravitreal injection of Ceftazidime and Amikacin. Vitreous tap was sent for gram stained, culture and sensitivity. Postoperatively, he was started empirically on IV Ciprofloxacin 160 mg BD, Guttae Ciprofloxacin, and Guttae Ceftazidime. Conjunctiva swab grew *Pasteurella canis* which was sensitive to all Beta lactams, Ciprofloxacin, Chloramphenicol, and Aminoglycoside. Post-operative was uneventful, absent signs of endophthalmitis or orbital cellulitis.

## 1. Introduction

The genera *Pasteurella* are small Gram-negative coccobacilli that are primarily natural habitant of the oral flora and digestive tract of various animals including domestic cats and dogs. However, these organisms can cause a variety of infections in humans. It is absent in the normal flora of the human [1]. There are several species of *Pasteurella* which can cause disease in humans such as *Pasteurella multocida *subsp. *multocida*, *P. multocida *subsp. *Septic, *and *P. canis *[2, 3]. Here we described a case of eye infection associated with *Pasteurella canis *following penetrating injury of the left eye. To the best our knowledge, *P. canis* is mainly reported as wound infection following dog bites without ocular involvement [4].

## 2. Case Report

A 3-year-old healthy boy presented with acute painful left eye associated with bleeding while playing with a broom stick made up from coconut skewers. There was history of cats as their pets but not dogs. Examination under anaesthesia of left eye revealed conjunctiva was injected and lacerated at the superonasal area. There was also presence of 2 mm full thickness scleral laceration with prolapsed vitreous at 10 o'clock which was 1 mm from the limbus. There were no RAPD, and anterior and posterior segment examinations were normal except for minimal vitreous haemorrhage. Visual acuity at presentation was 6/12 on the affected eye and 6/9 on fellow eye. Gram staining, culture, and sensitivity from the wound site were sent. Patient underwent scleral toilet and suturing with intravitreal tap followed by intravitreal injection of Ceftazidime and Amikacin. Vitreous tap was sent for gram staining, culture, and sensitivity.

Postoperatively, he was started on IV Ciprofloxacin 160 mg BD, Guttae Ciprofloxacin, and Guttae Ceftazidime 2 hours each and Ointment Chloramphenicol ON. VA on day 1 post operatively was 6/12. Initial gram staining from the wound site taken on the first day of injury yielded no pus cell and no organism detected. However culture yielded greyish colonies growth on blood agar. *Pastereulla canis *was identified through Vitek with 95% possibility. Sensitivity test done on MHBA revealed sensitivity to all Beta lactams, Ciprofloxacin, Chloramphenicol and Aminoglycoside. No colony growth was detected from vitreous tap specimen. IV Ciprofloxacin was continued until he completed 1 week and on D8 post-op he was discharged. Postoperative recovery was uneventful, B and scans and ocular examination showed no sign of vitritis or retinal detachment. Guttae prednisolone 1% QID was added and continued for 2 weeks. Visual acuity on upon discharge was 6/9. The wound was cleaned and signs of endophthalmitis and orbital cellulitis were absent. At 6 weeks after injury he showed full recovery with good vision of 6/9 by Cardiff chart which is equal to the fellow eye.

## 3. Discussion

Human infection with *Pasteurella *can be divided into three types: infection occurring after animal bites, usually from dogs or cats; infection occurring after other animal exposures; infection with no known animal contact [5]. Infection after animal bites is the most commonly reported clinical setting for the organism. In general dog bites are most common, followed by cat bites. In addition to bites, *Pasteurella *infections also have been reported after dog and cat scratches and from the licking of open wounds by these animals [4]. As in this case, infections occurred after penetration injury to the left eye by the coconut skewers used as a broom that probably has been contaminated by animals particularly cat. Further history revealed that patient's family kept cats as their pets but not the dogs. However, no confirmatory history obtained about the direct contact with the cat's faeces. This patient had been managed aggressively at the very early stage with topical, intravitreal, and parenteral antibiotic which most likely contributed to the absence of further complications.

*Pasteurella* infections of the ocular structures were caused by *P. multocida* in most reported cases. The types of ocular infections include conjunctivitis, keratitis, periocular abscess, periocular cellulitis, and endophthalmitis [6–8]. *P. canis* on the other hand mainly causes the soft tissue infection and wound infection [9]. Hoffman et al. reported *Pasteurella multocida* endophthalmitis in a 61-year-old male with no previous history of animal bites or scratch who was managed with vitrectomy and aggressive antibiotic including subconjunctival, parenteral, and intravitreal administration of ampicillin [6]. In our case, the organism isolated was *P. Canis* cultured from the swab at the wound which was not part of the normal flora of the eye [1, 4]. Absent signs of severe infection such as endophthalmitis or orbital cellulitis following isolation of *P. Canis* were most likely due to early and aggressive eradication of the organism with antibiotic.

Several virulence factors have been described in *Pasteurella *spp. Toxin production has been demonstrated in some *Pasteurella *isolates from animals. particularly, leukotoxin has been isolated from *Pasteurella haemolytica. *Leukotoxin is toxic to ruminant leukocytes and is thought to impair cellular response in lung tissue and to stimulate the inflammatory response [10]. In addition, most virulent *Pasteurella *strains produce polysaccharide capsules. Several decades of clinical experiences with *Pasteurella *and numerous in vitro studies indicate that penicillin is the best antimicrobial agent for the treatment of virtually all forms of infection [5]. The oral cephalosporins, cefuroxime, and cefixime along with parenteral agents including ceftriaxone and cefoperazone demonstrate excellent in vitro activity and are probably good substitutes for penicillin. Among non-*β*-lactam antibiotics, agents with in vitro activity include tetracyclines, fluoroquinolones, chloramphenicol, and trimethoprim-sulfamethoxazole [5].

Antimicrobial resistance among *Pasteurella *isolates is rarely reported in humans. Tetracyclines, erythromycin, and penicillin are most commonly associated with resistance. Penicillin-resistant strains have been isolated only from respiratory tract infections. Most animal-bite injuries can be treated with oral antimicrobials on an outpatient basis. Severe or partially responding infections may necessitate hospitalization and parenteral antimicrobial administration, along with surgical intervention [11]. More severe infections may require parenteral antibiotics. Intravenous ampicillin-sulbactam, ticarcillin-clavulanate, piperacillintazobactam, cefoxitin, and carbapenems (imipenem-cilastatin, meropenem, ertapenem) are excellent empiric options for animal-bite injuries, providing gram-positive, gram-negative, and anaerobic coverage. The new tetracycline-derivative tigecycline also has excellent in vitro activity against *P. multocida *and other pathogens encountered in animal and bite injuries. If *P. multocida *is the only isolated organism, therapy may be changed to intravenous penicillin G. Once clinical improvement is noted, oral penicillin VK is an option. Patients with penicillin allergies can receive minocycline, doxycycline, fluoroquinolones, trimethoprim-sulfamethoxazole, or azithromycin [11].

In this case patient was given empirical intravitreal antibiotics which were Amikacin and Ceftazidime. He was also given IV Ciprofloxacin BD for 1 week and Gutt Ciprofloxacin and Ceftazidime and occasional Gutt Chloramphenicol ON. Based on drug sensitivity test, the organism showed sensitivity towards all the antibiotics given. Perhaps, Immediate and prompt treatment that had been carried out for this young child prevented the spread of infection particularly endophthalmitis which may carry poor visual prognosis.

## 4. Conclusion

In conclusion, *Pasteurella canis *is rare but still a possible cause of eye infection especially following eye injury that has contact with soil in origin. Early treatment with empirical antibiotic may prevent further complication.
